# Diagnostic Utility of Synovial Fluid Cell Counts and CRP in Pediatric Knee Arthritis: A 10-Year Monocentric, Retrospective Study

**DOI:** 10.3390/children9091367

**Published:** 2022-09-08

**Authors:** Irene Nyaaba, Pierre-Yves Zambelli, Aziz Chaouch, Aline Bregou, İlker Uçkay, Eleftheria Samara

**Affiliations:** 1 Department of Pediatric Orthopaedics, University Hospital of Lausanne, 1011 Lausanne, Switzerland; 2 Centre Universitaire de Médecine Générale et Santé Publique, 1000 Lausanne, Switzerland; 3 Universitätsklinik Balgrist, 8008 Zürich, Switzerland

**Keywords:** knee arthritis, diagnosis, synovial fluid, leucocyte counts, CRP

## Abstract

Background: Orthopedic surgeons often use the intra-articular white blood counts (WBCs) and the percentage of polymorphonuclear cells (PMN) in the diagnosis of an acute swollen and painful knee joint in children. Today, there is no established threshold for the synovial WBC, and their differentiation, as indicative of native joint knee bacterial arthritis. We determine the sensitivity and specificity of synovial WBCs and PMN percentages in the prediction of a community-acquired, acute bacterial native joint septic arthritis (SA) in the pediatric population. Methods: A retrospective study on healthy children 0–16 years of age who underwent knee joint aspiration for a community-acquired, acute irritable knee effusion in our tertiary-care children’s hospital between May 2009 and April 2019 was conducted. We divided the study population into two groups according to the detection of bacterial arthritis in the synovial fluid (bacterial arthritis versus its absence) and compared the intra-articular leukocyte and C-reactive protein (CRP) levels. Results: Overall, we found a statistically significant difference regarding the total CRP (*p* = 0.017), leukocyte or PMN counts (*p* ≤ 0.001 in favor of a bacterial arthritis). In contrast, the percentage of the neutrophils was not determinant for the later confirmation of bacterial pathogens, and we were unable to establish diagnostically determining minimal thresholds of the intra-articular CRP and leukocyte levels. Conclusions: This pilot study suggests that either the leukocyte or PMN counts may be associated with a bacterial origin of knee arthritis in children. We plan a larger prospective interventional study in the future to confirm these findings including the investigation of other joint aspirate biomarkers.

## 1. Introduction

An acutely swollen and painful knee joint is a frequent emergency in a Pediatric Department. In healthy children, acute bacterial native joint septic arthritis (SA) needs to be considered as the first differential diagnosis. An accurate diagnosis is imperative to initiate treatment and probably to prevent further destruction of the cartilage. Generally, several predictive models and criteria have been established to distinguish between an SA and the more benign conditions, such as transient non-bacterial synovitis [1,2,3,4,5,6], viral arthritis, or other non-infectious affections, which would all influence the therapeutic approach.

Usually, the hallmark symptoms of SA are fever (>38.5 °C), acute joint pain and swelling, and a consecutive limited range of motion or pseudoparalysis [7]. As a general rule, clinicians perform a complete blood cell count with the differential assessment of other serum inflammatory markers such as the C-reactive protein (CRP). In the case of local important inflammation in the knee, they continue with an arthrocentesis and send the liquid for Gram staining and bacterial cultures together with an intra-articular leukocyte count; a targeted polymerase chain reaction (PCR) analysis for *Kingella kingae* in children less than 48 months age; or a PCR for Lyme disease if there is clinical concern. Magnetic resonance imaging (MRI) is the gold standard exam for the suspicion of osteoarticular infection in children.

Among all these investigations, the joint aspiration results guide the definitive therapeutic choice. In cases of uncertain clinical diagnosis, orthopedic surgeons often use the synovial WBC and the percentage of PMNs to determine the correct bacterial diagnosis of SA. However, there is no established threshold for the minimal WBC as indicative of native joint SA in healthy children. In adults, values close to, or greater than, 100,000 cells/mm^3^ are suggestive of SA, whereas values of less than 25,000 cells/mm^3^ in the synovial fluid are highly unlikely to be caused by a bacterial infection. Several authors have questioned this threshold and its clinical utility [4,8,9,10]. Similarly, some studies have used an intrasynovial PMN percentage cut-off of >90% to be indicative of SA [11]. This threshold, firstly established in the literature by Ward et al. in 1960 [12], does not reflect the reality in joint aspirations in recent studies [13,14,15]. Despite its routine use in the work-up of patients with suspected SA, there is little research on the predictability of local synovial values in diagnosing SA in children. In this study, we determined the sensitivity and specificity of knee synovial WBC and the percentage of PMNs in the diagnosis of community-acquired bacterial SA in the pediatric population. We chose these two parameters because both are relatively cheap, already established in daily clinical life, rapid, barely influenced by prior systemic antibiotic therapy, ubiquitously available, or easy to obtain.

## 2. Patients and Methods

We retrospectively reviewed the data from all patients under 16 years of age who underwent knee joint aspiration due to an acute, community-acquired irritable knee effusion in our tertiary-care children’s hospital between May 2009 and April 2019 (Ethical Committee Approval CER-VD 2021-01762). We concentrated on primary, monoarticular, community-acquired knee arthritis cases. The exclusion criteria were: immunocompromised individuals, children already diagnosed with rheumatologic disease, patients with acute knee effusion following an open fracture or trauma, nosocomial cases after surgery or infiltration, tuberculosis, Lyme disease, polyarticular involvement, secondary SA due to remote infection (e.g., endocarditis), and chronic or subacute knee effusions. We assessed the demographic characteristics (age, sex, and laterality); the historic time points (duration of symptoms, history of prior antibiotics, and history of recent illness); the radiographic or US evidence of effusion; and the results of laboratory analyses such as serum white blood cell count (WBC) and CRP levels on admission. The synovial fluid CRP and the white blood cell count with the PMN percentage in repartition were documented. The synovial fluid Gram staining was not examined since it is a poor screening tool for the detection of SA and is particularly ineffective for the detection of Gram-negative organisms [16]. The joint fluid was sent to the laboratory for immediate inoculation to blood agar (incubated under anaerobic conditions), chocolate agar (incubated in a CO2-enriched atmosphere), CDC anaerobe 5% sheep blood agar (incubated under anaerobic conditions), and brain–heart broth. In the case of systemic signs of inflammation, we sampled a total of four bottles for blood cultures from the peripheral veins. Our institute used the BD BACTEC FX automated blood culture system. Two PCR assays were also used for bacterial identification when the standard cultures were negative. The initial aliquots were stored at −80 °C until they were processed for DNA extraction. A universal broad-range PCR amplification of the 16sRNA gene was performed using BAK11w, BAK2, and BAK533r primers. In children less than 48 months old, we also performed the specific real time PCR assay targeting the *K. kingae* gene’s rtx toxin in the synovial fluid and on the oropharyngeal specimens [17]. For this study, we censored the results of intraoperative tissue analyses in case of a consecutive surgical lavage. All samples were taken within six hours of the patient’s arrival to the Emergency Unit and transported to the Bacterial Laboratory of our university center within twelve hours.

## 3. Statistics

We summarized the numerical variables by their median and range and compared across the two diagnostic groups using a Mann–Whitney U test. Additionally, we used a univariate logistic regression to model the probability of SA stratified upon the synovial WBC count, the synovial PMN count, or the percentage of intra-articular PMNs. We determined minimal thresholds by visually plotting the results against the confirmed diagnosis of bacterial SA. The in-sample predictive performance of each model investigated the area under curve (AUC) of the Receiver Operating Characteristic (ROC). A *p*-value < 0.05 (two-tailed) was considered as statistically significant. We performed all analyses with R [18].

## 4. Results

From the initial cohort of 64 children who underwent knee joint aspiration for suspicion of acute SA, 14 patients were excluded as they met at least one of our exclusion criteria. Twenty patients were further excluded from the population cohort due to a lack of synovial fluid leukocyte counts. The final cohort of 30 patients was then categorized in two groups: the confirmed septic arthritis (SA) group (*n* = 17) with positive bacterial fluid cultures; and the control group (others: O; *n* = 13) with the absence of bacteria. This control group also included the negative broad-range PCRs and PCRs for *K. kingae* in the synovial fluid. The positive for oropharyngeal PCR *K. kingae* patients with negative synovial cultures (including broad range PCR and *K. kingae*) were included in the O group and considered as carriers [19].

The children were aged between 1 and 184 months with a median age of 43 months. In the SA group, the median age was 27 months, and it was 87 months in the O group (*p* = 0.675). The overall median synovial fluid WBC was 14.7 with an overall median proportion of PMNs of 79%.

Within the SA group, nine patients had *K. kingae*, four had *Staphylococcus aureus*, two had *Streptococcus pyogenes*, one had *Escherichia coli* and one child revealed *Streptococcus mitis*. From a microbiological point of view, only one patient had a positive blood culture from *Staphylococcus aureus*. *K*. *kingae* was only recovered from classic synovial cultures in two cases. In seven cases, the pathogen was revealed by a *K. kingae*-specific real-time PCR assay performed in the synovial fluid. Table 1 compares the basic demographic results between the SA and O groups. We detected statistically higher values regarding the intrasynovial CRP (*p* = 0.017), the WBC, and the PMN counts (*p* ≤ 0.001). In the SA group, the synovial fluid WBCs oscillated between 0.95 and 581 cells × 10 ^9^/L (G/L) with a median value of 70.4. The median PMN% was 82%. The median CRP value was 36.0 mg/L. In the O group, the median synovial WBC was 5.0 G/L with a median PMN% of 73%. The CRP values were between 3.5 mg/L and 44 mg/L with a median value of 8.0 mg/L. Table 2 reports the odds ratio quantifying the effect of each predictor on the probability of SA defined by a positive culture and as the corresponding AUC. Unlike the overall percentage of intra-articular PMN, both the WBC and PMN counts had a statistically significant effect on the probability of SA. The AUC of the logarithm of the WBC counts and PMN counts was also relatively high at 0.86 and 0.88, respectively, which highlights the high discriminative power of these predictors of SA.

## 5. Discussion

Our study focused on the accuracy of the intrasynovial WBC and PMN counts in the differentiation of bacterial, community-acquired SA from other causes of native knee joint inflammation in children. We revealed a positive correlation. The children with bacterial SA yielded significantly higher intrasynovial inflammatory markers than the control group. In contrast, the proportion of PMNs was not discriminatory between the study groups, and we failed to detect a minimal threshold in the absolute synovial counts concerning the synovial markers, making their practical use less reliable in daily clinical settings. Similar results were found for the CRP values. Hence, the synovial markers remain a laboratory element which is supportive for clinical decision making but is not determinant on its own. Although in the “other” (O) group we did not detect a causative pathogen in either the standard cultures of synovial fluid or in the PCR, we may have also included septic arthritis from other fastidious microorganisms involved in osteoarticular infections which have not yet been recognized as pathogens, and further efforts should be made to develop new molecular techniques to identify them [17,20,21].

Besides its retrospective nature, our study faces several additional limitations. First, we had a small sample size of only 30 cases, which forced us to only consider the linear effects for the predictors of interest as more complex functional forms would have required more data. Additionally, due to the limited sample size, we could not investigate the effect of potential confounders such as age and causative pathogens, nor adjust for the case-mix in multivariate analyses. Second, the predictive performance of each model (quantified by the AUC) was assessed on the same sample that served to define the model. In order to confirm the ability of each predictor of interest to reliably predict SA, a future study should evaluate the predictive performance of these predictors in a new, prospective, and ideally larger sample that does not serve for model definition.

Third, other joint aspirate biomarkers such as the intrasynovial CRP test via ELISA assay, alpha-defensin immunoassay levels, and lactate and leucocyte esterase levels [22,23] could potentially solidify the suspicion of septic arthritis in children and should be tested in future studies with children. In the adult population, apart from the standard synovial inflammatory markers (CRP, WBCs, and PMNs) other joint biomarkers are used as useful tools for the detection of septic arthritis and interestingly as forms of rapid tests, such as the Synovasure. The lateral flow immunoassay Synovasure TM (Zimmer, Warsaw, IN, USA) is a rapid diagnostic test that detects alpha-defensin (Synovasure TM) in joint fluid, thus ensuring the prompt diagnosis of prosthetic joint infection [24]. To our knowledge, this test has never been tested in a pediatric population with the suspicion of septic arthritis.

Lastly, the study criteria required microbiologically proven bacterial arthritis and can only apply to our study population, i.e., knee SA in healthy children without prior antibiotic use before the arrival to the Emergency Unit. For a pilot study in a community setting, this requirement is acceptable. In contrast, our results would probably be more biased than for other SA populations such as nosocomial cases, adults, or chronic arthritis. In the literature regarding adult and nosocomial patients, a substantial number of clinical bacterial native joint arthritis episodes remain culture-negative even when the samples have been taken intraoperatively [25].

Despite its limitations, this retrospective study is one of the few studies concentrated on the knee joint effusion of pediatric patients and the potential distinguishing parameters between septic and non-septic arthritis [26,27,28,29]. Kocher predictive criteria for the hip joint are not applicable for the prediction of septic knee arthritis [26], and neither is the modified Kocher–Caird [2,30]. However, in recent studies, the combination of two specific factors (the inability to bear weight and an elevated C-reactive protein) was found to be strongly predictive [2,30]. As an alternative to the original Kocher criteria, Baldwin et al. found that by increasing the number of criteria they achieved higher predictive values which included: pain with an arc of motion below 30 degrees, CRP level >4.0 mg/L, notion of episodes of fever, and an age below 2 years were all positive predictive values which were able to help distinguish septic arthritis of the knee from non-septic forms such as Lyme arthritis [27]. In Lyme-disease-endemic areas, Deanehan et al. suggested a septic arthritis prediction model including only two laboratory criteria, which had the same sensitivity and a higher specificity than the published Kocher criteria. Children with knee effusion who have a peripheral blood absolute neutrophil count <10 × 10 ^3^ cells/mm^3^ and an erythrocyte sedimentation rate <40 mm/hour are at low risk for septic arthritis and, in the right clinical context, may not require diagnostic arthrocentesis [28]. The same authors concluded in another study that in Lyme-endemic areas, synovial fluid results alone do not differentiate septic from Lyme arthritis [29]. In the same way, one can assume that apart from clinical and paraclinical parameters, the addition of synovial fluid measurements could strengthen the positive predictive value for septic arthritis of the knee.

To sum up, our pilot study suggests that the use of either synovial WBC counts or synovial PMN counts, two easily accessible and cost-free parameters, are useful for the diagnosis of SA in children. According to the current literature, the combination of clinical and paraclinical measurements are not accurate enough to distinguish septic knee arthritis from other causes of knee effusion in the pediatric population; therefore, one can assume that the incorporation of synovial inflammatory markers after arthrocentesis would help in a surgeon’s definitive decision of treatment. Based on the results of Obey et al. [26], the combination of the measurement of PCR in the blood and PCR in the synovial fluid may be of predictive interest. Rapid tests of the combined measurement of synovial fluid biomarkers such as alpha-defensin, lactate, and leucocyte esterase would be of special interest in future prospective studies in pediatric septic arthritis. Given that the *K*. *kingae* is the dominant causative pathogen [17,31,32,33,34] in 6–48 month-old children, the development of a rapid oropharyngeal and synovial PCR test would be of great interest as well.

We plan a larger prospective study in the future where our findings will be tested, as well as other synovial biomarkers. Multi-centric studies are also encouraged to distinguish not only septic vs. non-septic arthritis, but also distinguish pyogenic from fastidious organisms. Basic research to develop a rapid PCR oropharyngeal and synovial test for the detection of *K*. *kingae* is also strongly recommended since such a positive test would guide pediatric orthopedic surgeons to an initial minimally invasive treatment, giving its high predictive specificity [35].

## 6. Conclusions

Acute knee septic arthritis is the most frequently observed joint infection in children. Rapid and accurate diagnosis is the prerequisite of an excellent evolution without sequelae. The modified Kocher–Caird criteria only have limited utility in predicting septic arthritis of the knee. This study shows that leucocyte and synovial PMN counts may be associated with a bacterial origin of knee arthritis in children. Thus, we believe that a combination of clinical, paraclinical, and synovial measurements can provide stronger positive predictive value for the diagnosis of septic arthritis.

## Figures and Tables

**Table 1:** Sample characteristics (median and inter-quartile range (IQR)) in confirmed septic arthritis (C-SA) and other (O) groups, with statistical significance of between-group differences assessed using the *p*-value of a Mann–Whitney test. C—reactive protein CRP, white blood counts WBC count, polymorphonuclear cells PMN (%) and PMN count are from synovial fluid.

Variable	C-SA Group Median [IQR]	O Group Median [IQR]	Group Comparison *p*-Value (Mann–Whitney)
Age (months)	27 [16–145]	87 [21–102]	0.675
CRP (mg/L)	36 [13–64]	8 [7–29]	0.017
WBC count (G/L)	70.4 [15.6–96.7]	5.0 [0.8–8.1]	<0.001
PMN (%)	82.0 [75.5–88.5]	63.3 [73.0–88.0]	0.321
PMN count * (G/L)	48.6 [26.7–89.2]	4.8 [4.3–8.4]	0.001

* derived as WBC count × PMN%.

**Table 2:** Odds ratio and area under the curve (AUC) with 95%CI quantifying the effect of each predictor on the probability of (septic arthritis) SA (univariable models). CRP, WBC count, PMN (%) and PMN count are from synovial fluid.

Predictor	Odds Ratio (95% CI)	AUC (95% CI)
WBC count	1.58 ^(1)^ (1.17–2.49)	0.86 (0.73–1.00)
PMN%	1.22 ^(2)^ (0.90–1.73)	0.63 (0.34–0.91)
PMN count *	1.55 ^(1)^ (1.10–2.58)	0.88 (0.72–1.00)

(1). Refers to the impact of doubling the value of the predictor, (2). refers to the impact of a 10% increase in PMN%, * derived as WBC count × PMN%.

## Data Availability

Data available on request due to restrictions of CER-VD (Ethical Committee Approval CER-VD 2021-01762). The data presented in this study are available on request from the corresponding author.
